# Amitriptyline-induced ventricular tachycardia: a case report

**DOI:** 10.1186/s13104-017-2615-8

**Published:** 2017-07-14

**Authors:** Khandker Mohammad Nurus Sabah, Abdul Wadud Chowdhury, Mohammad Shahidul Islam, Bishnu Pada Saha, Syed Rezwan Kabir, Shamima Kawser

**Affiliations:** 1 Dhaka Medical College Hospital, Dhaka, Bangladesh; 2 Anwer Khan Modern Medical College Hospital, Road-08, Dhanmondi, Dhaka, 1209 Bangladesh; 3 Dr. Sirajul Islam Medical College & Hospital Ltd, Dhaka, Bangladesh

**Keywords:** Tricyclic antidepressants, Amitriptyline overdoses, Amitriptyline-induced ventricular tachycardia, Wide complex tachycardia

## Abstract

**Background:**

In Bangladesh, each emergency physician faces amitriptyline overdose nearly a day. An acute cardiovascular complication, one of the worst complications is mainly responsible for the mortality in tricyclic overdose. Recently, we managed ventricular tachycardia in a young female presented with an impaired consciousness 10 h after intentionally ingesting 2500 mg amitriptyline. Here, we report it, discuss how the electrocardiography is vital to acknowledge and predict it and its’ complications and also the recent update of the management of it.

**Case presentation:**

A young married Bangladeshi-Bengali girl, 25-year-old, having a history of disharmony with her husband, came with an impaired consciousness after intentionally ingesting 2500 mg amitriptyline about 10 h before arrival. There was blood pressure 140/80 mmHg, heart rate 140 beats-per-min, temperature 103 °F, Glasgow coma scale 10/15, wide complex tachycardia with QRS duration of 178 ms in electrocardiography, blood pH 7.36. Initially, treated with 100 ml 8.4% sodium bicarbonate. After that, QRS duration came to 100 ms in electrocardiography within 10 min of infusion. To maintain the pH 7.50–7.55 over the next 24 h, the infusion of 8.4% sodium bicarbonate consisting of 125 ml dissolved in 375 ml normal saline was started and titrated according to the arterial blood gas analysis. Hence, a total dose of 600 mmol sodium bicarbonate was given over next 24 h. In addition to this, gave a 500 ml intravenous lipid emulsion over 2 h after 24 h of admission as she did not regain her consciousness completely. Afterward, she became conscious, though, in electrocardiography, ST/T wave abnormality persisted. So that, we tapered sodium bicarbonate infusion slowly and stopped it later. At the time of discharge, she was by heart rate 124/min, QRS duration 90 ms in electrocardiogram along with other normal vital signs.

**Conclusion:**

Diagnosis of amitriptyline-induced ventricular tachycardia is difficult when there is no history of an overdose obtained. Nevertheless, it should be performed in the clinical background and classic electrocardiographic changes and wise utilization of sodium bicarbonate, intravenous lipid emulsion, and anti-arrhythmic drugs may save a life.

## Background

In Bangladesh, amitriptyline is the commonly used drug as an anti-depressant and overdose cases are faced almost daily in our practical life, though there are no accurate data regarding the incidence and prevalence of it. Worldwide, tricyclic antidepressants (TCA) are identified as one of the most frequently ingested substances in self-poisoning hitherto after being first reported in 1959 [1]. Among them, amitriptyline is one of the toxic tricyclic antidepressant [2–5]. Fatal dysrhythmias are rare as ventricular tachycardia occurs to only approximately 4% of all cases [6, 7]. Here, we report a case of amitriptyline-induced ventricular tachycardia (AIVT) and discuss the importance of electrocardiography (ECG) in this case and the recent update of overall management of it.

## Case presentation

### Case

A 25-year-old young married Bangladeshi-Bengali female was brought to the emergency department by her relatives with the presentation of an impaired level of consciousness after intentionally ingesting 100 × 25 mg (2500 mg total) tablets of amitriptyline approximately 10 h prior to arrival. There was no suicide note written but found 10 empty strips, each containing 10 tablets that indicated the patient had taken 100 tablets of 25 mg amitriptyline. She had a history of disharmony with her husband. 2 years backward, she incised her left forearm by blade deliberately.

**Fig. 1:**
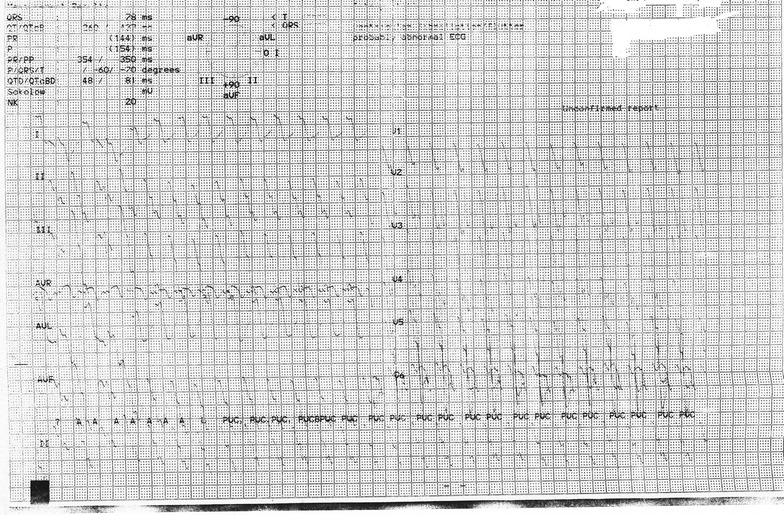
Ventricular tachycardia following intentional ingestion of 2500 mg amitriptyline in a female patient on admission

**Fig. 2:**
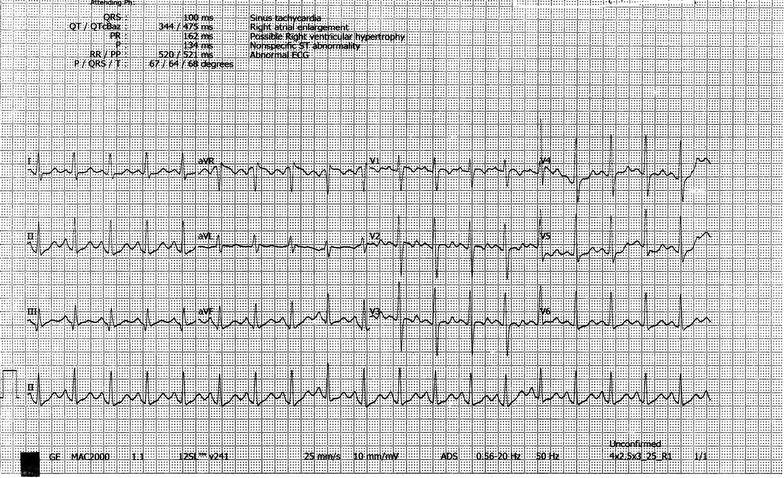
Sinus tachycardia after giving NaHCO_3_ with QRS duration 100 ms and ST/T wave abnormality

**Fig. 3:**
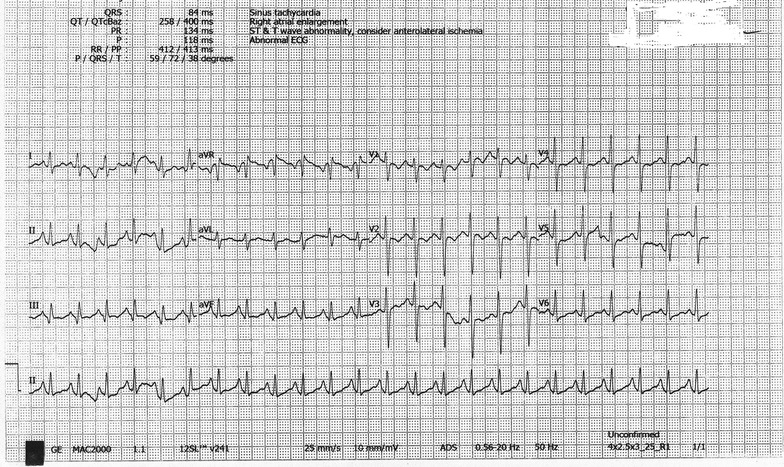
On day 2, electrocardiography showed sinus tachycardia with ST/T wave abnormality in chest leads

At the emergency, blood pressure was 140/80 mmHg, heart rate 140 beats-per-min, respiratory rate 24 breaths/min with oxygen saturation 95% on room air, temperature 103 °F, Glasgow coma scale 10/15 as she opened his eyes to verbal response, localized painful stimuli and spoke inappropriate words (E2, M5, V3). Abdominal examination revealed distended urinary bladder. There was no lateralizing sign in the neurological examination. ECG revealed wide complex tachycardia (Fig. 1) with QRS duration 178 ms. Blood pH was 7.36. She immediately transferred to a cardiac coronary unit (CCU) for monitoring and management purpose, where she was treated initially with 100 ml 8.4% sodium bicarbonates in an undiluted state given intravenously over 5 min. Heart rate back to 115 beats-per-min within 10 min after infusion of sodium bicarbonate. In the limb leads, QRS duration came to 100 ms and in chest leads, ST/T wave changes were seen on ECG (Fig. 2). Over the next 24 h, sodium bicarbonate infusion consisting of 125 ml dissolved in 375 ml normal saline was started and titrated according to the report of arterial blood gas analysis done 6 h to maintain the pH 7.5–7.55. Initially, sodium bicarbonate infusion started at a rate of 100 ml/h. A total dose of 600 mmol sodium bicarbonate was required in 24 h. Other investigations—serum electrolyte, random blood sugar was normal. On Day 2, as she did not regain his consciousness, intravenous lipid emulsion 500 ml was given over 2 h after 24 h of admission. Afterward that, she regained her consciousness completely. Sodium bicarbonate infusion was tapered slowly and stopped it later. Her serum electrolytes done at the end of Day 2 were also normal. In ECG, ST/T wave abnormality persisted (Fig. 3). On Day 3, she was transferred to post-CCU and evaluated by a psychiatrist. On Day 4, she was discharged with following heart rate 124/min with QRS duration in the limb lead 90 ms in ECG along with other normal vital signs.

## Discussion

In Bangladesh, amitriptyline is a most commonly used drug among the tricyclic antidepressants. It is lipophilic and has a large volume of distribution. It is primarily metabolized by the liver. Most active metabolites of amitriptyline are nortriptyline and 10-hydroxynortriptyline [8]. In overdoses, amitriptyline exerts its effects [9–11] by the following way:

1. Inhibition of presynaptic neurotransmitter reuptake (norepinephrine and serotonin).
2. Blockade of the cardiac fast sodium channels.
3. Antagonism of central and peripheral muscarinic acetylcholine receptors.
4. Antagonism of peripheral alpha-1 adrenergic receptors.
5. Antagonism of histamine (H1) receptors.
6. Antagonism of central nervous system (CNS) gamma-aminobutyric acid (GABA)-A receptors.
7. Blocks the hERG human cardiac potassium channels.

In therapeutic doses, the elimination half-life of amitriptyline and its metabolites is 19–72 h [8]. In toxic doses, it is average 37 h, and commonly greater than 60 h [12, 13] as pharmacokinetics differs from the therapeutic pharmacokinetics of amitriptyline in many ways [14]:

1. Due to anticholinergic effects, gut motility decreases. It delays the absorption of amitriptyline.
2. Metabolic pathways become saturated. It reduces the first pass metabolism resulting in increased bioavailability.
3. Overdoses cause respiratory depression resulting in acidaemia. It increases the amount the free drug due to effects on plasma glycoprotein binding.
4. Significant enterohepatic recirculation prolongs the final elimination.

Prolonged toxicity was also described in a CYP2D6-deficient patient as CYP2D6 is responsible for the metabolism of amitriptyline and its metabolites metabolism [15].

In amitriptyline overdose, the progression of clinical toxicity is unpredictable. Acute ingestion of >5 mg/kg causes significant cardiovascular, CNS and anticholinergic symptoms [16]. With it, the reported lowest dose of amitriptyline that causes severe toxicity in a young adult is 300 mg [17].

Acute cardiovascular toxicity is primarily responsible for the mortality in amitriptyline overdose. Sinus tachycardia (rate 120–160 beats/min in an adult) occurs due to inhibition of presynaptic norepinephrine reuptake and anticholinergic action [10]. Amitriptyline also inhibits the fast sodium channel in the His-Purkinje system and myocardial. As a result, depolarization of the cardiac action potential is slowed and propagation of depolarization though both myocardial and conducting tissue is delayed. Ultimately, it decreases conduction velocity, increases the duration of repolarization and prolongs absolute refractory period [10, 18]. It also blocks the hERG human cardiac potassium channel which contributes to arrhythmogenic side effects of amitriptyline [11]. In ECG, prolongation of the PR, QRS and QT intervals, nonspecific ST-segment and T-wave changes, atrioventricular block, right axis deviation of the terminal 40 ms vector of the QRS complex in the frontal plane (T 40 ms axis) and the Brugada pattern (downsloping ST segment elevation in leads V_1_–V_3_ in association with right bundle branch block) is seen [19–23]. In several studies, several sensitive indicators are found to identify and risk-stratify the patients with cyclic antidepressant.

1. A T40-ms QRS axis between 120^0^ and 270^0^: It is 8.6 times more common in TCA poisoning than that in non-TCA poisoning. In practical life, without specialized computer-assisted analysis, a T40-ms axis is not well quantified. An abnormal terminal rightward axis can be estimated by noting a negative deflection (terminal S wave) in lead I and aVL and a positive deflection (a terminal R wave) in lead aVR [24, 25].
2. The maximal limb leads QRS complex duration: QRS duration greater than 100 ms is associated with an increased incidence of serious toxicity, including coma, the need for intubation, hypotension, seizures and dysrhythmias [26].
3. Terminal R wave and R/S ratio in aVR: The amplitude of terminal R wave in equal or more to 3 mm and R/S equal or more to 0.7 in aVR are also sensitive in predicting seizures and dysrhythmias [26].

Fatal dysrhythmias are rare as ventricular tachycardia (VT) and ventricular fibrillation (VF) occur in only approximately 4% of all cases [6, 7]. It happens most often in patients with prolonged QRS complex duration and/or hypotension. Hypoxia, acidosis, hyperthermia, seizures and β-adrenergic agonist may also predispose to it [27].

Hypotension during an overdose occurs due to decreased contractility of the myocardium and peripheral vasodilatation due to antagonism of peripheral alpha-1 adrenergic receptors. Refractory hypotension during TCA overdose is the most common cause of death [28].

Altered mental status and seizures are the primary manifestations of CNS toxicity. Lethargy, alterations in consciousness, coma is the usual consequence. It is due to both antihistamine and anticholinergic effects, whereas seizure occurs as a result of antagonism of GABA-A receptor in the brain by TCA [29, 30]. Some studies have indicated that the level of consciousness of presentation is the most sensitive clinical indicator of complications after a TCA overdose [20, 31, 32].

In the management of a patient with AIVT, initially, the key issue is securing the airway, breathing, and circulation. After that, sodium bicarbonate should be used to treat the AIVT, even in the absence of acidosis before using any anti-arrhythmic drugs. To assess the use of sodium bicarbonate in AIVT, several studies were done [33–35]. One guideline recommends it for the treatment of AIVT [36]. The use of sodium bicarbonate in AIVT gives some benefit [33, 37–39].

1. It provides high sodium load for greater sodium entry into the myocardial cells in the presence of sodium channel blockade by the amitriptyline.
2. It alkalinizes the blood resulting in reduction of efficacy of the amitriptyline for the sodium channel leading to reduction of TCA binding to sodium channel.
3. Alkalization of the blood reduces the unbound amitriptyline and increases the protein binding. It ultimately serves to counteract sodium channel blockade in the myocardium by amitriptyline.
4. Alkalization of the blood also prevents acidosis, which exacerbates the cardiac toxicity.
5. The sodium and the bicarbonate both act synergistically to increase their individual effects.

The initial dosage is 1–2 mEq/kg IV bolus in AIVT [34, 35, 40]. There is no specific recommendation about the timing of repeat dose. It is wiser to wait at least 10 min. In this case, AIVT reverted within 10 min of the 1st dose of sodium bicarbonate. After that, a continuous IV infusion of sodium bicarbonate will be started to maintain a blood pH 7.50–7.55 [41]. It is better to taper the bicarbonate therapy before discontinuing it after the clinical improvement of conscious level and improvement, but not necessarily normalization of abnormal ECG findings. In the meantime, the clinical and lab parameters should be monitored closely as volume overload, hypokalemia, hypernatremia, and metabolic alkalosis resulted from prolonged sodium bicarbonate therapy [42–44]. ECG finding as sinus tachycardia may persist for up to 1 week following ingestions [45].

Treatment with magnesium sulphate may be considered in AIVT. Several studies are, however, done to assess its value in the treatment of AIVT [46–48]. One randomized controlled trial recruited a total of 72 patients with TCA overdose examining the efficacy of magnesium sulphate on patients of TCA overdose in mean duration of hospital stay and mortality rates between the group treated with bicarbonate infusion only and the group treated with bicarbonate infusion plus infusion of magnesium sulphate revealed that reduced hospital stays (p <0.001) and reduced mortality rate (p = 0.052) observed in group treated with infusion of bicarbonate plus magnesium sulphate [49]. One guideline recommends the usage of magnesium sulphate in cases of refractory dysrhythmias causing hemodynamic instability [36].

Anti-arrhythmic agents—Class IA (quinidine, procainamide, disopyramide), Class IC (flecainide, propafenone) and Class III (amiodarone, sotalol) are contraindicated as Class IA, IC prolongs the depolarization like amitriptyline and Class III prolongs the QT interval, which predisposes to develop arrhythmias [10].

Though the use of intravenous lipid emulsion (ILE) in the treatment of toxicity of lipophilic drug overdose is limited to a few case reports and one randomized controlled trial. Realizing the benefit outweighs the hazard it was used in this instance when the patients’ point of consciousness did not improve after 24 h of sodium bicarbonate infusion. The precise mechanism of ILE is not fully realized. The original theory is well-known as “lipid-sink”; nowadays it is supplanted with the more exact term “lipid shuttle” mechanism as it exercises its effects by three mechanisms—(1) lipid scavenging of the drugs (2) a volume effect (3) the cardiotonic effects. It is found in a study of combining dose–response to the effects of ILE in bupivacaine-poisoned rats with PBPK/PD modeling [50–53]. In an amitriptyline overdose or poisoning, probably ILE acts in like style as both amitriptyline & bupivacaine has a similar pharmacological weak base peak 9.4 capable of accepting a proton to become cationic [54]. In this example, bicarbonate infusion before ILE administration may increase the percentage of circulating amitriptyline in an unionized state and augment the efficacy of the ‘lipid-shuttle’ as in acidosis, bupivacaine demonstrated a lower bupivacaine-lipid binding capacity [55, 56]. As far we know, in all case reports of amitriptyline overdose treated with ILE, sodium bicarbonate was infused concomitantly with ILE. The outcome was good [57–65]. Probably bicarbonate-induced alkalosis may increase amitriptyline-lipid binding capacity in whole instances. One randomized control trial included a total of 108 cases examining the efficacy of ILE on duration of cardiotoxicity and subsequent complications of severe TCA showed that there is no statistically significant difference was observed between the groups received standard treatment with sodium bicarbonate and standard treatment plus intravenous lipid emulsion concerning the time needed for ECG reversal, in blood pressure at the time of ECG reversal, in mortality and in length of hospitalization. Though it also noted that time needs for ECG reversal 20 min shorter in the ILE group. The complete article is still not put out and not assessed to check the quality of evidence [66]. To determine the role of acid/base status on the potential efficacy of ILE therapy on amitriptyline overdose, more work is needed. Though the causal relation to ILE infusion is not clear, reports of complications in adults are transient lipemia, pancreatitis, acute respiratory distress syndrome (ARDS) and post-ILE cardiac arrest. Recently the clinical toxicology’s lipid emulsion collaborative work group said that the quality of evidence is low to very low, but they didn’t remark on ‘not the use of ILE in amitriptyline overdose’ [67].

After being reverse the dysrhythmias, is there any role of plasmapheresis, hemodialysis, and hemoperfusion? Recently, EXTRIP Workgroup recommended not to performing extracorporeal treatment (ECTR) in patients with TCA poisoning [68]. Even afterward the utilization of high-cut off (HCO) dialyzer, the eliminated amount did not surpass 3% of the total ingested dose per dialysis session reported in one case report [69].

In TCA overdose, all major complications occur within 6 h of ingestion [70]. Though the incidence of late complication is really low, ventricular arrhythmias had been reported up to 5 days after ingestion [71–73]. All patients should receive cardiac monitoring until the ECG has become normal. An alert and orientated patient with QRS duration less than 100 ms in the ECG is the best indicator for safe transfer to a medical or psychiatric ward for psychiatric evaluation and discharge after gastrointestinal (GI) decontamination and a minimum observation of 6 h [32, 74].

## Conclusions

AIVT is one of the devastating complications of an amitriptyline overdose. Sometimes, diagnosis of AIVT is very hard as there is no history of an overdose obtained due to an altered story of awareness. Diagnosis should be performed along the foundation of clinical grounds and classic ECG changes. Anti-arrhythmic drugs should be warded off in this example. Thus, wise use of medication may save a life.

## Abbreviations

AIVT: amitriptyline-induced ventricular tachycardia
ARDS: acute respiratory distress syndrome
CCU: cardiac coronary unit
CNS: central nervous system
ECG: electrocardiography
ECTR: extracorporal treatment
GI: gastrointestinal
HCO: high-cut off
HP: hemoperfusion
ILE: intravenous lipid emulsion
TCA: tricyclic antidepressant
VT: ventricular tachycardia
VF: ventricular fibrillation
